# Temporal dynamics and uncertainty in binaural hearing revealed by anticipatory eye movements

**DOI:** 10.1121/1.5088591

**Published:** 2019-02-04

**Authors:** Matthew B. Winn, Alan Kan, Ruth Y. Litovsky

**Affiliations:** 1 Department of Speech-Language-Hearing Sciences, University of Minnesota, 164 Pillsbury Drive SE, Minneapolis, Minnesota 55455, USA; 2 Waisman Center, University of Wisconsin-Madison, 1500 Highland Avenue, Madison, Wisconsin 53705, USA

## Abstract

Accurate perception of binaural cues is essential for left-right sound localization. Much literature focuses on threshold measures of perceptual acuity and accuracy. This study focused on supra-threshold perception using an anticipatory eye movement (AEM) paradigm designed to capture subtle aspects of perception that might not emerge in behavioral-motor responses, such as the accumulation of certainty, and rapid revisions in decision-making. Participants heard interaural timing differences (ITDs) or interaural level differences in correlated or uncorrelated narrowband noises, respectively. A cartoon ball moved behind an occluder and then emerged from the left or right side, consistent with the binaural cue. Participants anticipated the correct answer (before it appeared) by looking where the ball would emerge. Results showed quicker and more steadfast gaze fixations for stimuli with larger cue magnitudes. More difficult stimuli elicited a wider distribution of saccade times and greater number of corrective saccades before final judgment, implying perceptual uncertainty or competition. Cue levels above threshold elicited some wrong-way saccades that were quickly corrected. Saccades to ITDs were earlier and more reliable for low-frequency noises. The AEM paradigm reveals the time course of uncertainty and changes in perceptual decision-making for supra-threshold binaural stimuli even when behavioral responses are consistently correct.

## I. INTRODUCTION

Binaural hearing refers to an intricate set of mechanisms whereby the auditory system compares acoustic information arriving at the two ears, and uses that information to perform important tasks, including localizing sounds (e.g., Mills, 1960; Macpherson and Middlebrooks, 2002) and understanding speech when there is background noise (e.g., Bronkhorst and Plomp, 1988; Hawley *et al*., 2004). The absolute accuracy and acuity of the binaural system has been studied in numerous ways, using a variety of behavioral techniques. In this study we apply an eye-tracking method to measure binaural sensitivity, drawing inspiration from eye-tracking studies of spoken word recognition.

As noted by Huettig and Altmann (2011), eye-tracking paradigms have had a transformative effect on the field of speech perception, allowing experimenters to observe how participants rapidly update their perception with incoming information (Tanenhaus *et al.*, 1995) and usually demonstrate some consideration of competing response choices even if uncertainty would not be indicated by behavioral responses (McMurray *et al*., 2008). Additionally, saccades indicate that perceptions can be rapidly revised (sometimes multiple times) before behavioral responses are given (Pickering and Traxler, 1998; Altmann and Kamida, 1999). Numerous eye-tracking studies indicate that perceptual judgments are typically not all-or-none, but instead involve some granular amount of certainty that evolves rapidly over time as the stimulus is heard—even when stimuli are easily categorized or well above an expected threshold.

Accuracy, precision, and acuity of the binaural system have been studied in a variety of ways, typically with behavioral responses like pointing, button-pressing, or neural response data in non-human animals. These are gold-standard measurements that have advanced the field and formed the bedrock of knowledge of the binaural system. However, it is reasonable to suspect that the granular sensitivity of eye-tracked responses would exceed that of behavioral responses in psychoacoustic studies, given the plethora of speech perception studies where such a pattern has prevailed. There is surprisingly little application of eye-tracking methods in basic psychoacoustics but potentially much to gain.

The auditory cues under investigation in this study are interaural level differences (ILDs) and interaural timing differences (ITDs), which are the primary auditory cues in the horizontal plane. For listeners with typical hearing, perception of ITDs is largely dependent on the frequency of the sound, with lower-frequency sounds requiring just dozens of microseconds of timing disparity to achieve reliable left or right perception (Klumpp and Eady, 1956). Perception of ITDs in higher-frequency sounds (i.e., above roughly 1500 Hz) is much less reliable for pure tones because of the breakdown of phase locking and because the wavelength of the sound is smaller than the size of the head, resulting in ambiguous phase between the ears. It is thus common to describe ITD as an interaural *phase* difference. However, ITDs are also perceptible not just in the temporal fine structure of a sound, but also in its amplitude envelope. So-called “envelope ITDs” can be used to lateralize sound (Henning, 1974; Neutzel and Hafter, 1976; Wightman and Kistler, 1992), although not as strongly or reliably as the corresponding cues in lower-frequency sounds. For ILDs, changes as small as 1 dB are reliably perceptible (Mills, 1960; Domnitz and Colburn, 1977) with neural measurements showing comparable responses across the entire audible frequency range (Jones *et al*., 2015), consistent with the naturally broad distribution of frequencies where ILDs emerge in the natural world (Młynarski and Jost, 2014).

Relatively little work has been done in perception of *changes* in binaural cues in continuous sounds, which is explored in the current study. Blauert (1972) tested detection of changes in ILDs that were sinusoidally modulated, but limitations of that study were noted by Grantham (1984), who posited the distinction between perception of specific directional changes versus non-specific “differences.” Grantham showed that ILD changes become less perceptible as they are more rapidly alternating and also when they are carried by lower-frequency noises. The relative weakness of binaural change detection was also shown by Stecker and Brown (2012), who used a train of Gabor pulses to explore perception of binaural cues at multiple timepoints during a stimulus. They found that ILD sensitivity was poorer at the middle of the sound compared to the onset and offset. Perception of fluctuations in ITDs has been tested somewhat more often, and modeled by Dietz *et al.* (2008) based on sensitivity to phase differences encoded by the lateral superior olive. Behaviorally, ITD changes are rather difficult to update after a listener perceives and perseverates a lateralized ITD at the onset of a sound (called “onset dominance,” cf. Franssen, 1960; Stecker and Bibee, 2014).

In the current study, we are interested in how perception of suprathreshold binaural cues is updated and rapidly solidified during exposure to a changing stimulus. This theme is motivated by three observations. First, most sounds in the everyday environment do not demand threshold-level sensitivity. We are rarely in situations where the absolute limits of the system are actually in play, and it is not clear whether perception of binaural cues above threshold is simply a transformation of performance at threshold. The second motivation is that nearly all auditory signals outside of the laboratory are temporally dynamic in nature. Sounds do not all instantaneously appear and reappear in different locations; they move. Finally, studies of other auditory abilities like word recognition and sentence processing show that listeners momentarily consider alternative perceptions before revising and committing to a final decision (Allopenna *et al*., 1998; Altmann and Kamide, 1999; McMurray *et al*., 2002). Perception might thus be described more appropriately as a process that unfolds over time, rather than only as a final decision or discrete event, as in a task where listeners are asked to identify the position of a sound source. The current study will investigate whether this framework holds true for perception of basic auditory properties like binaural cues. Below, we introduce some of the background from other fields of study that bring potentially relevant considerations for the study of binaural perception.

### A. Eye tracking in auditory and linguistic sciences

Eye tracking is a useful method to investigate perceptual processes that unfold over time because multiple eye movements can be recorded in a single trial and are rapid enough to be time-locked to stimulus events (Altmann and Kamide, 2007). Eye movements (saccades) are more metabolically efficient than other behavioral movements like button-pressing, mouse clicking, finger pointing, or spoken responses. Saccades should therefore be less affected by individual differences in gross motor control and more likely to provide a more accurate measure of the time it takes to make a perceptual decision. Saccades are measurable across the lifespan, and are particularly popular in measures of pre-linguistic children. Like evoked potentials, eye gaze is valuable for so-called *online* responses; a listeners' gaze is tracked through the course of a whole stimulus without the listener needing to stop to report momentary updates on perception. Importantly, saccades are also known to alternate rapidly between multiple options in an informative way that might not emerge in behavioral responses, as described below.

In cases where perception is subject to competition between alternative judgments, eye movements can show the degree of commitment or consideration of various responses before a final decision is made—even in the case of stimuli whose behavioral responses are entirely consistent. McMurray *et al.* (2002) and McMurray *et al.* (2008) have demonstrated this principle using speech stimuli where acoustic cues gradually varied between those for /b/ or /p/ at word onset. Listeners heard the stimuli and clicked on images whose labels varied by this consonant contrast (e.g., “bear” and “pear” or “beach” and “peach”). If stimuli were near the acoustic-perceptual boundary between /b/ and /p/ categories, listeners were more likely to have spent more time looking at the image other than the one they ultimately selected with their behavioral response. That is, eye-gaze patterns indicated that stimuli near the b-p category boundary elicited perceptual competition between both potential responses. In this and other studies, even stimuli considered to be unambiguous (because they are consistently labeled with the same behavioral response) elicit looks to competitors at a rate that is above zero, and which tend to update over time to ultimately reflect the final choice, even if the initial gaze was toward the competitor response. Even though it might not be intuitive to directly compare speech categorization and more fundamental auditory abilities like sound lateralization, the common feature that we highlight is that there is an acoustic dimension that has a level that divides the potential auditory input into categories, be they two phonemes or a division between left and right.

Patterns of gaze fixations can reflect continuous accumulation of evidence over time in auditory perception (Allopenna *et al*., 1998), which can be linked to specific earlier- or later-occurring information in a stimulus (McMurray *et al*., 2017), and observed well before any behavioral response is made. Reinisch *et al*. (2010) showed, for example, that as the word “sentimental” is spoken, listeners will visually fixate equally on the correct target word and also the word “centimeter”; it is not until the contrastive penultimate syllable is heard that eye gaze toward the incorrect word is suppressed in favor of the correct target word. This shows that even though the listener could ultimately get the answer correct, some time was spent considering the alternate answer. The value of this approach is that it demonstrates that the final outcome of a response choice does not by itself indicate the various incremental stages of processing that led to the ultimate decision. In language sciences, this has spawned entire fields of study, whose progress might be leveraged in the auditory sciences as well.

While the present study is focused on psychoacoustic perception rather than language processing, some key principles remain especially relevant: auditory information is accumulated and processed rapidly, and perceptual judgments can be updated and changed multiple times between candidate options before a behavioral response is recorded. By using time-varying measures of perception such as eye movements, we can obtain information about how perception evolves during exposure to a stimulus.

The specific measurement technique that we use in the current study was originally developed for studies of auditory and visual categorization in infants. The *anticipatory eye movement* (AEM) paradigm is essentially a conditioning procedure where observers see an object whose rightward or leftward movement is associated with different auditory categories (McMurray and Aslin, 2004). For example, a ball moves across the computer screen and is temporarily occluded behind another object. It is programmed to emerge on the left side whenever the listener hears “da” and on the right whenever “ba” is heard. After learning this association, observers hear one of those two syllables in the test trial, and anticipate the emergence of the ball on the correct side; we can verify their auditory perception via their AEMs toward the correct side of the occluder before the ball emerges. In the current study, we are dealing with simple leftward and rightward auditory motion, which can be naturally linked with leftward or rightward motion of the visual object without explicit conditioning. As such, we expect that the magnitude of inter-individual and within-individual variability will not be constrained by the strength of conditioning, and that the method, if successful, would be suitable for testing participants across a wide range of ages (cf. McMurray and Aslin, 2004, who tested infants as young as 6 months old) and physical abilities (i.e., those who might have limited arm/hand mobility).

### B. Hypotheses and predicted value

The primary goal of the study is to demonstrate that the methods used to calculate the degree of certainty and rapid changes in decision-making in phonetic/psycholinguistic perception can be applied to basic psychoacoustic stimuli. We seek additional knowledge about suprathreshold perception that would parallel what has been learned about perception of linguistic sounds. To justify the choice of this eye-tracking method, which is more costly and complicated than standard behavioral methods, we set out to test whether there are aspects of perception that can be revealed by eye movements that specifically would have not emerged with the use of behavioral methods. These include rapid updates of perceptual decisions related to resolution of perceptual competition and quickness of revisions and granular differences in the processing of suprathreshold stimuli. We hypothesize that when listeners are less certain about their perceptions (e.g., when cue magnitudes are small or when ITDs are carried by high-frequency noises), we should see not only longer reaction times for saccades, but also more variability in the willingness to commit to a decision, resulting in more revisions of gaze direction and more time spent looking at the wrong answer even if the ultimate choice was correct. With increasing certainty, we should observe earlier and more steadfast judgments and quicker revisions in the case of initial wrong-way fixations.

We offer two primary reasons why the suprathreshold time-series measures in this study are important. First, while behaviorally acquired just-noticeable differences (JNDs) provide information about the smallest difference we can discriminate, it remains unknown whether processing of larger cue magnitudes is saturated at maximum performance levels, can be predicted by JNDs, or is nonlinear in some other way. Second, eye gaze reveals information about the time course of processing—not only how quickly a response is reached, but the interim stages of considering alternative options. Prior literature suggests strongly that these intermediate states of perception are not reliably detectable through behavioral responses, which might reasonably be called “final decisions.” However, correct and incorrect responses for sounds near a listener's threshold are not necessarily all-or-none decisions; they might involve some weighted decision process that accumulates information over time in a way that illuminates sub-processes at play. Even though stimuli used in classic binaural experiments do not contain the syntactic, lexical, or phonetic ambiguities that are hallmarks of psycholinguistic studies, there is still a likelihood that non-speech auditory perception involves some amount of uncertainty. Classical transformed up-down behavioral methods (cf. Levitt, 1971) inherently account for the inconsistency of all-or-none responses, and there could be value in investigating within-trial activity to complement blocked-average scores. As sensory neurons operate stochastically in intermediate or probabilistic states of activation, granular measures, such as eye tracking, might help reveal some of the underlying activity that might otherwise be lost in some standard methods.

## II. METHODS

### A. Participants

A total of 43 adult listeners (28 women) between the ages of 18 and 35 yr (mean age: 20.2 yr) participated in these experiments. Of this group, 26 participated in the ILD and 22 participated in the ITD conditions; 5 listeners participated in both conditions. Each listener was screened for normal hearing thresholds [less than or equal to 20 dB hearing level (HL)] at octave frequencies between 250 Hz and 4000 Hz. Participants were ineligible if they presented with threshold differences greater than 10 dB difference across ears at the same frequency. Participants were not excluded on the basis of language background, but all denied having any hearing difficulty or learning disability. One listener was excluded because he was suffering from lack of sleep, and another listener was excluded because of difficulty in understanding directions. Two other listeners were excluded because they could not reliably respond to the stimuli judged to be easily perceptible for a typical normal hearing listener (16 and 24 dB changes in ILD, or 500 and 750 *μ*s changes in ITD for 500 Hz noises). All participants gave informed content on a protocol approved by the Internal Review Board (IRB) at the University of Washington, where all testing was performed.

### B. Stimuli

Stimuli consisted of 4.8-s-long videos showing a ball moving vertically through a Y-shaped pipe with accompanying audio of a narrowband noise that contained either a change in ITD or ILD during the presentation as determined by the movement of the ball. Samples are available in the supplementary materials 6–12.^1^ Below we describe the details of the auditory and visual components of the videos.

### C. Auditory component

Auditory stimuli consisted of 1/3-octave narrowband noises at three center frequencies of 500, 1500, and 4000 Hz. These bands were generated using the Praat software (Boersma and Weenink, 2015) by generating Gaussian white noise and then applying a Hann filter in the frequency domain.

For ILD stimuli, the left and right channels were generated independently to create “uncorrelated” noises. The goal was to avoid any discernible ITD cue in these stimuli, relegating listeners to use the ILD to complete the task. We speculated that the 0 *μ*s ITD of an interaurally correlated noise stimulus might pull perception toward the center, weakening ILD perception. It should be noted, however, that Brown and Tollin (2016) provided convincing evidence that the extent of perceptual lateralization based on ILD is actually rendered *more* accurate by interaural correlation. This aspect of the stimuli will be examined later in Sec. IV B.

At the midpoint of each stimulus (at time 2.4 s), the ILD changed from 0 to 2, 4, 8, 16, or 24 dB in either the left or right direction. There were also stimuli that had no systematic changes in ILD, apart from momentary interaural disparities in the envelopes because of interaural decorrelation. All changes in level were applied using a cross-fading procedure such that the first half ended with a 30 ms decay time and the second half began with a 30 ms rise time. Those corresponding 30 ms transition periods were overlapped and added with each other to produce a smooth transition with no perceptible change in overall intensity.

For ITD stimuli, a single noise token was generated and duplicated for each ear so that the temporal fine structure and envelope were perfectly correlated across ears. Stimuli began as diotic signals and then at the midpoint either stayed diotic or changed to have an ITD of 63, 125, 250, 500, or 750 *μ*s in either the left or right direction via a full-waveform shift. All ITD stimuli contained a full-depth amplitude modulation at midpoint with 15 ms decay out of the first half and 15 ms rise time into the second half. This amplitude modulation was applied in order to prevent unwanted artifacts (clicks) or gaps in the fine structure that could affect listener responses. This step was not taken in the ILD stimuli, where whole-waveform multiplication was applied at a zero crossing to avoid artifacts.

### D. Visual component

**FIG. 1.**
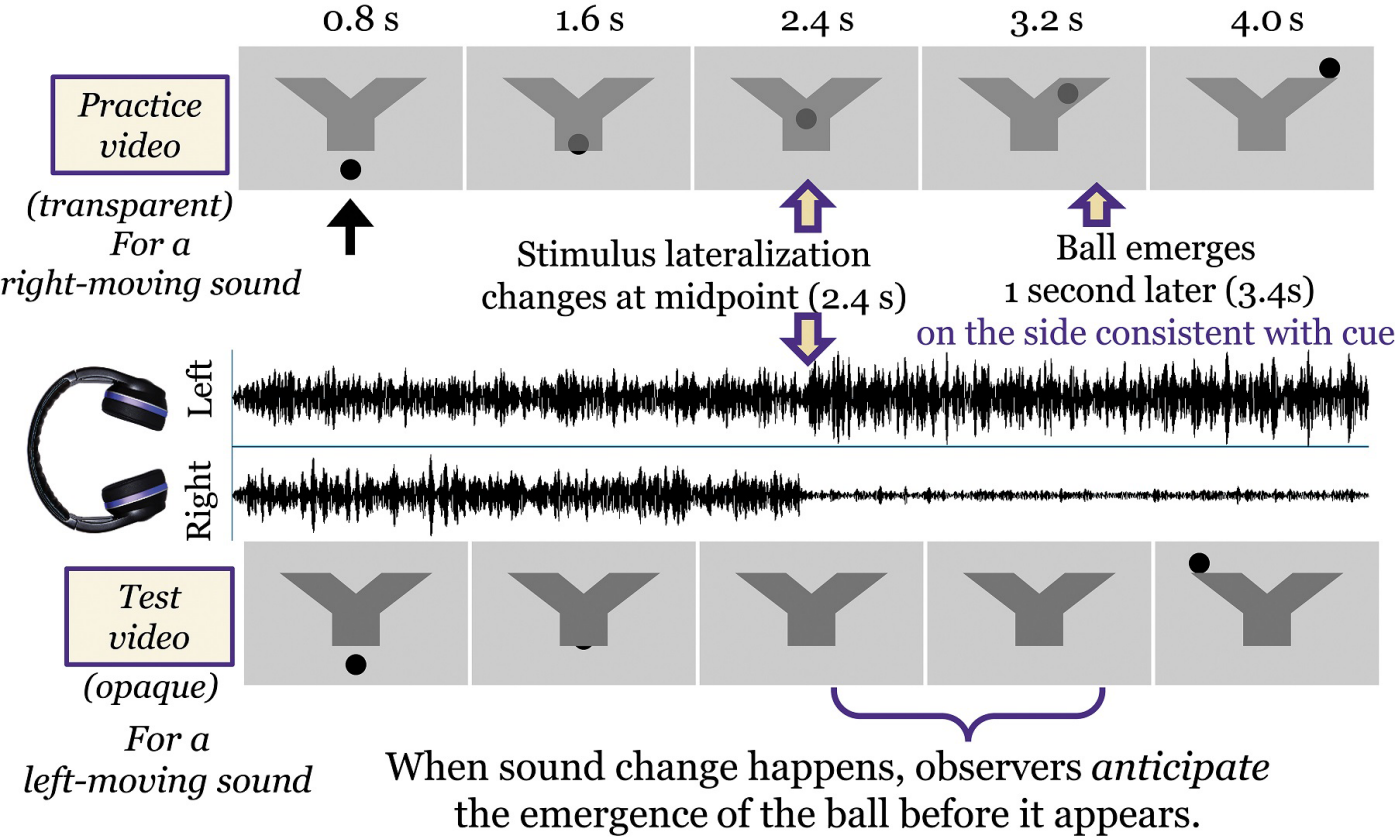
(Color online) Trial timeline, showing screenshots of video for the practice (upper images, with transparent pipe) and test (lower images, with opaque pipe) conditions for a right-moving and a left-moving auditory condition, respectively. The ball moves at a constant speed through the pipe, emerging from either the left or right branch at the top. In the middle of this figure, an ILD stimulus is shown, in which the left channel intensity is increased and the right channel intensity is decreased. The leftward auditory motion is paired with a leftward motion of the ball. The change in auditory cue occurs when the ball would be exactly midway through its upward trajectory as it begins to move left or right.

The visual component of the stimuli was a black ball moving smoothly through a gray Y-shaped pipe that was centered on the screen (Fig. 1). The ball entered the screen from the bottom at the center and proceeded upward through the pipe to emerge from either the left or the right side. Left- and right-lateralized auditory stimuli (defined by their ILD or ITD levels) were always paired with matching video stimuli with congruent motion of the animated ball. The video frames were timed such that the change in ILD or ITD cue was concurrent with the ball reaching the center of the pipe. The next frame following the cue change was the first movement of the ball to the left or right. The emergence of the ball at the upper left or right extension of the pipe occurred exactly 1 s after the onset of the cue change (i.e., 1 s after the midpoint of 2.4 s).

There were two versions of the audio-visual stimuli. The practice version featured a semi-transparent pipe where the motion of the ball was visible throughout the video. This served to acclimate the participants to the speed of the ball and the general concept of the congruence of auditory and visual cues. Practice videos always featured a 16 or 24 dB ILD cue because those were predicted to be the easiest to perceive and thus easiest to establish the general pattern of the stimuli.

The test version of the stimuli featured an opaque pipe, where the motion of the ball was entirely obscured from the moment it entered the bottom of the pipe to the moment it emerged at the upper left or right extension. In this version of the stimuli, the one second between auditory cue change (i.e., change from ILD or ITD of zero to some non-zero value) and the emergence of the ball was the time during which the participant directed her or his gaze to the side of the pipe indicated by the audio.

It should be noted that we deliberately chose to use a Y-shaped pipe rather than the triangle-shaped occluder used by McMurray and Aslin (2004). This is to more strongly encourage a “left” or “right” response from the participants without a viable “center” option. However, it was not a forced-choice task, *per se*, as a participant could (and frequently would) change their decision at any time prior to the ball appearing visually, but sometimes not make any choice until the answer appeared visually.

### E. Procedure

Participants were seated in a chair in a double-walled sound-attenuated room (Acoustic Systems RE-243, Austin, TX). Each listener had her/his head comfortably stabilized using the SR Research (Ontario, Canada) chin rest, which ensures steady positioning of the face on the eye tracker camera image. The height of the chin rest was adjusted until comfortable. Prior to the experiment, listeners were provided with a verbal explanation of the audiovisual stimuli and task. Listeners were told that “The ball moves in sync with the sound. Your task is to listen for the sound change and predict where the ball will emerge from the pipe as soon as you hear the sound move left or right. Some sounds will be difficult to perceive, but give your best guess as soon as you know.” Auditory stimuli were delivered through Sennheiser HD600 headphones (Wedemark, Germany), and visual stimuli were shown on a Dell (model P2214HB, Round Rock, TX) 24-inch monitor positioned approximately 60 cm away from the face. The experiment was programmed in matlab (The MathWorks, Natick, MA) and used the PsychToolbox Version 3.0.12 (Brainard, 1997; Pelli, 1997; Kleiner *et al*., 2007), which contains EyeLink toolbox functions used to handle triggering of events with the eyetracker (SR Research Eyelink 1000).

Understanding the AEM paradigm is exceedingly easy for listeners, as shown in previous studies where infants performed with no explicit verbal training (McMurray and Aslin, 2004). Each participant in this study was familiarized with the task using eight practice videos before moving on to the test conditions. Apart from the small number of aforementioned participants who were not able to reliably respond, participants generally considered the task to be relatively natural and straightforward.

Test stimuli were randomized for noise frequency and cue magnitude, so there was no indication of the level of difficulty for any upcoming stimulus. Repetitions of each of the 36 unique stimuli (3 noise frequencies × 6 cue magnitudes × 2 directions) were interleaved in a random order. Each stimulus was repeated between seven and nine times. Increases from seven to nine were used for those participants who were considered at risk for data loss because of noisy gaze tracking. Trials were automatically paced, but every six trials, the playback was paused until the participant advanced the experiment with the press of a keyboard button; this allowed for shifting of posture or rest of the eyes in between runs of trials. Longer breaks were given once every 12–15 min. Total testing time was roughly 45–50 min.

### F. Analysis

#### 1. Binning and coding of the eye-gaze data

The scoring of saccades was binned into discrete categories of correct and incorrect (based on the stimulus), not based on the exact gaze position (e.g., slightly left, left, *far* left), for three reasons. Primarily, the Y-shaped occluder had a fixed target place for the emergence of the ball, meaning the saccade should have been to a specific gaze target regardless of stimulus cue magnitude. Furthermore, greater excursions of gaze direction would not necessarily indicate better perception. Should a stimulus change be perceptible very quickly, as in the case of an easy 24 dB ILD, the saccade might have been smaller in magnitude as the ball should be not very far from the center of the pipe by the time the change is noticed, and the gaze might be directed at where the ball is “hidden.” Conversely, if the participant waits longer to make a saccade, the estimated position of the ball should be farther away from the center since it has been “moving” behind the occluder in the interim. Alternatively, the participant could have decided to gaze only at the pipe endpoint and not the intermediate points. Greater *magnitude* of gaze excursion was therefore not a consistent or informative outcome measure. Future work might explore the degree of gaze displacement as an indicator of auditory perception where specific screen targets can be used for greater confidence in the answer.

Looks to the correct and incorrect sides were coded as “+1” and “−1,” respectively. Fixations that remained in the center were coded as “0.” This scheme was chosen to effectively “penalize” incorrect judgments and code them distinctly differently than lack of judgment. See supplementary material 1 for further discussion of the method for saccade detection and timing.^1^

#### 2. Quantification of data features

The time-series data reflecting success rate over time were inspected for trends in latency and slope. Data were fit using a three-parameter sigmoid to explicitly allow the upper asymptote to vary as a function of stimulus attributes like frequency and cue level. The intercept (i.e., horizontal position of psychometric functions) and slope were also free to vary. Latency to 50% criterion performance was extracted from the data and compared across stimulus parameters. Additionally, we counted numbers of saccades and the distribution of saccade times across the duration of the trial.

## III. RESULTS

### A. Overview of eye-tracking results

**FIG. 2.**
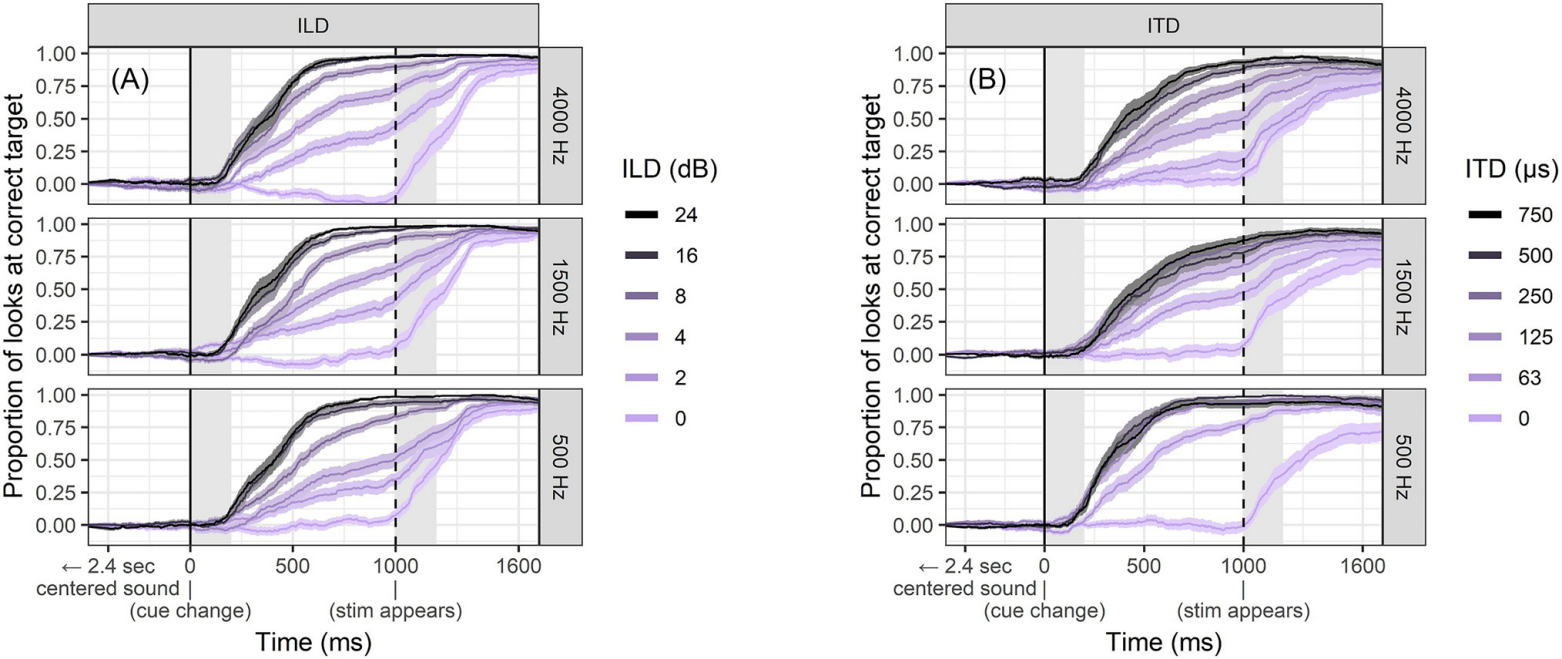
(Color online) Success rate of looking to the correct side over time, consistent with the laterality of an ILD (left panels) or ITD (right panels). Each timepoint is an average of the number of correct (+1), incorrect (−1), and no-response (0) categories collected across the trials. The left shaded area indicates the portion of time between 0 and 200 ms post cue onset, which is generally considered to be too fast to execute a saccade. The right shaded areas denote the portion of time between 0 and 200 ms after the visual cue to the true answer appeared on screen. Ribbon width represents ±1 standard error of the mean.

**FIG. 3.**
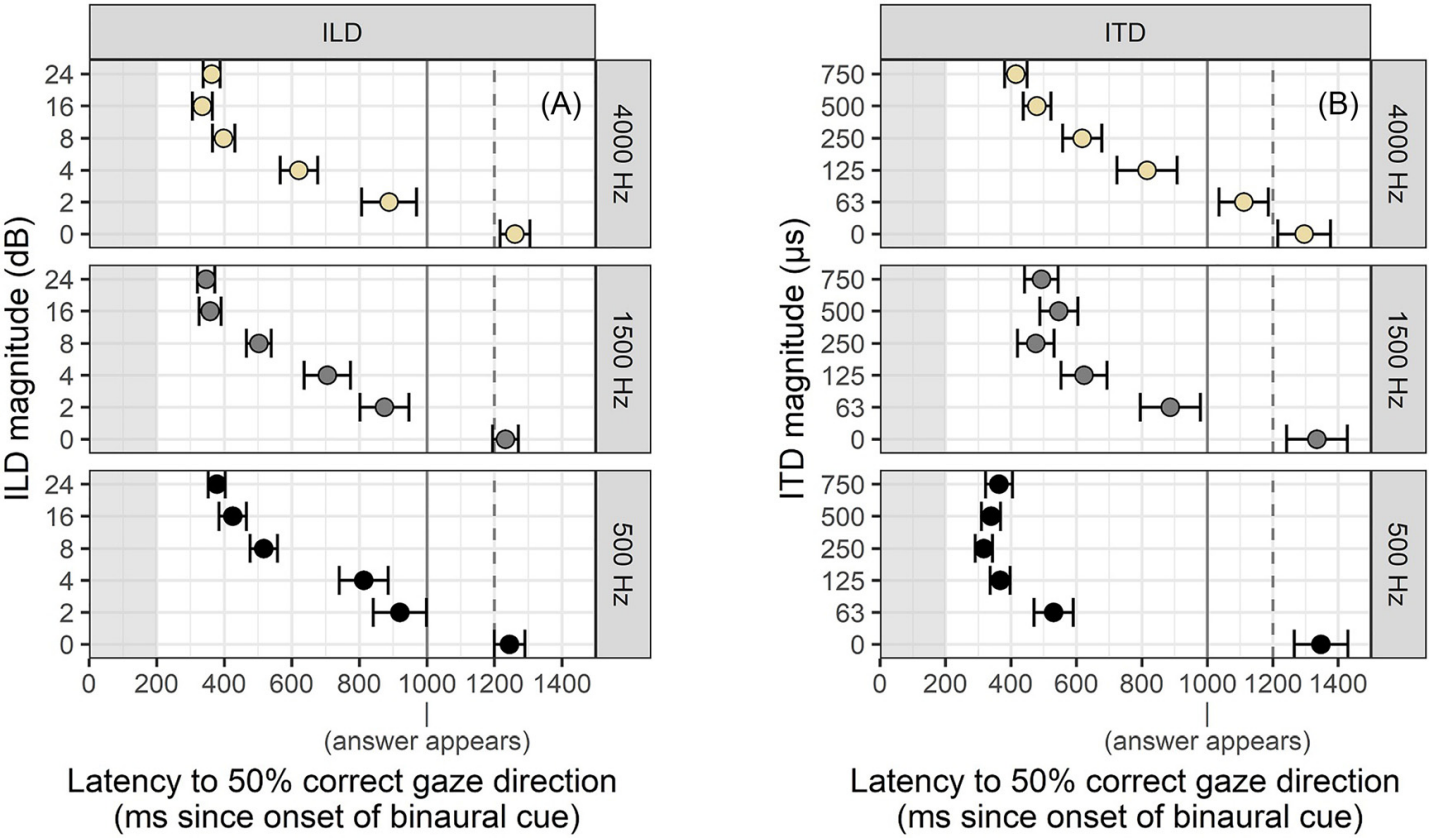
(Color online) Latency to achieve 50% average success rate for ILD and ITD perception. This represents the time at which the lines in Fig. 2 cross the 50% threshold. Error bars represent ±1 standard error of the mean.

As expected, participants directed their gaze to the side indicated by the change in binaural cue. Figure 2 shows the likelihood of looking at the correct target over the timeline of the trial. It can be clearly seen that there is a gradually increasing likelihood of earlier gaze fixations with increasing ILD across all frequencies. In other words, responses were not simply reflective of threshold, but instead reflective of the suprathreshold magnitude (i.e., saliency) of the ILD cue. For very easily perceptible cues such as 16 or 24 dB ILD, success rate increased rapidly starting at roughly 200 ms, which is as fast as could be expected, given that it is roughly the amount of time needed to simply program and execute a saccade. Responses to smaller-magnitude ILDs were often initiated at around the same time, but did not reach the same level of performance until later (as will be shown in Fig. 3), since gaze direction was not always correct.

In contrast to the ILD results, the effect of ITD changes in low-frequency 500 Hz noise bands were less gradual across stimulus magnitude; all ITD magnitudes greater than 63 *μ*s elicited equally fast response toward the target direction. Conversely, for high-frequency 4000 Hz noise bands, saccade speed and success rate were driven more by cue magnitude, and always slower than perception of the same ITD magnitude in low-frequency stimuli. Recall that the well-documented onset dominance effect should limit the listener's ability to update her or his ITD lateralization when the cue change occurs in the middle of the stimulus; these stimuli contained a full-depth amplitude modulation at the moment of cue change that likely promoted the good performance measured here.

The results of the curve-fitting analysis are displayed in Table I and illustrated in the supplementary materials 2 and 3 (verifying close fit to the raw data).^1^ Using these parameters, one can estimate the time where listeners generally achieved any particular threshold level of performance. For simplicity, the time corresponding to 50% performance is listed in the last column in Table I.

**TABLE I.** Parameter estimates for the sigmoidal functions describing the time-series data in Fig. 2. The form of the function is performance = asymptote/(1 + exp(−slope ∗ (time − shift))). Applying this function to a time range spanning 2–3.5 (s) would reproduce the basic form of the data. The rightmost column is the time where the function is estimated to cross 50%. NA indicates that the function does not cross 50% within the time window used for modeling (from 400 ms before stimulus onset to 1100 ms post stimulus onset).

Frequency (Hz)	ILD (dB)	Slope	Asymptote	Shift	Midpoint estimation
500	2	5.128	0.414	3.025	*NA*
500	4	8.134	0.517	2.944	3.36
500	8	7.133	0.847	2.902	2.953
500	16	10.105	0.927	2.798	2.814
500	24	9.402	0.979	2.796	2.801
1500	2	2.727	0.869	3.395	3.506
1500	4	6.558	0.687	2.929	3.079
1500	8	9.471	0.87	2.886	2.918
1500	16	9.382	0.95	2.781	2.792
1500	24	9.928	0.978	2.76	2.765
4000	2	7.231	0.457	2.984	*NA*
4000	4	7.76	0.716	2.873	2.981
4000	8	9.636	0.883	2.793	2.821
4000	16	9.343	0.97	2.75	2.757
4000	24	10.493	0.972	2.763	2.768
Frequency (Hz)	ITD (μs)	Slope	Asymptote	Shift	Midpoint estimation
500	63	8.627	0.77	2.858	2.929
500	125	9.057	0.936	2.75	2.765
500	250	10.71	0.934	2.727	2.74
500	500	11.283	0.968	2.727	2.733
500	750	11.126	0.923	2.729	2.744
1500	63	7.52	0.483	2.921	*NA*
1500	125	7.366	0.692	2.884	3.014
1500	250	7.868	0.821	2.821	2.877
1500	500	8.016	0.779	2.843	2.916
1500	750	8.75	0.854	2.833	2.872
4000	63	5.277	0.375	3.28	*NA*
4000	125	6.531	0.535	2.922	3.329
4000	250	7.655	0.753	2.915	3.004
4000	500	8.506	0.863	2.828	2.866
4000	750	8.25	0.928	2.811	2.83

### B. General summary of saccade timing and success rate

Figure 3 illustrates the latency to reach 50% correct saccade performance across all cue magnitudes in the three noise center frequency conditions. There was a general pattern of lower latency for larger ILD magnitude for all noises with 16 and 24 dB ILD responses reaching criterion at roughly 400 ms and 2 and 4 dB ILD responses reaching criterion at roughly 600–900 ms post cue onset (note: these numbers include the 200 ms necessary to execute a saccade after perceiving the sound). For 4 and 8 dB ILD stimuli, there was a shorter latency of responses to high-frequency noises. This result was not specifically hypothesized; there is a possibility that this effect is due to other factors in the stimuli apart from noise center frequency, which will be clarified further in Sec. IV B.

Latency to reach 50% performance for ITD stimuli varied widely across frequency. Consistent with the presumed dominance of low-frequency fine-structure ITD relative to relatively weaker envelope ITD, the low-frequency stimuli elicited the quickest performance with all ITDs above 63 *μ*s reaching criterion within 300–350 ms (including 200 ms saccade overhead time). Latency was shorter for low-frequency ITDs by about 100 ms compared to the lowest latency for the high-frequency ITD stimuli. Performance for the mid-frequency 1500 Hz ITD stimuli was intermediate to that for the low- and high-frequency stimuli. Responses to high-frequency 4000 Hz noise varied dramatically as a function of cue magnitude with fastest responses (400 ms) to 750 *μ*s cues and responses to 125 *μ*s cues around 800 ms. Figure 3 suggests that criterion latency for 63 *μ*s cues for 4000 Hz stimuli was roughly 1100 ms, but as will be discussed below in Sec. III F, deeper analysis of the data reveals a slightly more nuanced story.

Analyses of variance (ANOVAs) were conducted to determine main effects of cue magnitude, stimulus frequency, and the interactions between those two terms on the latency to 50% correct gaze direction separately for ILD and ITD stimuli.

For ILD stimuli, there was a statistically detectable main effect of cue magnitude (*F* = 193; *p* < 0.001) and a marginally detectable effect of stimulus frequency (*F* = 2.934; *p* = 0.054). Follow-up *t*-tests were conducted to further analyze differences between latencies to stimuli that appeared to be subtly different according to Fig. 3 (i.e., those comparisons that were not so obvious from the data and thus required statistical testing). There were statistically detectable differences between latencies for 4 dB and 8 dB ILDs for all three stimulus frequencies (*t* = 3.38 for 500 Hz; *t* = 2.72 for 1500 Hz; *t* = 3.4 for 4000 Hz, all *p* < 0.01). Results were similar for comparisons between 8 dB and 16 dB ILDs for 4000 Hz and 1500 Hz stimuli, although the difference in latency for 8 dB and 16 dB ILDs for 500 Hz (*t* = 2.21; *p* = 0.032) would not survive significance criterion adjustment for multiple comparisons.

Stimulus frequency affected the responses reliably for ILD values in the middle of the test range. Latencies for 4000 Hz were shorter than those for 500 Hz for 4 dB ILDs (*t* = 1.99; *p* = 0.051) and for 8 dB ILDs (*t* = 2.69; *p* < 0.05). Other comparisons were made between 4 dB and 8 dB ILDs for 500 Hz and 1500 Hz and between 1500 Hz and 4000 Hz, but no statistical differences emerged.

For ITD in 500 Hz noise stimuli, the only statistically detectable difference in latency to 50% correct was between 63 *μ*s and any of the other stimulus magnitudes (*t* = 2.64; *p* < 0.05 for comparison of 63 and 125 *μ*s). The same pattern emerged for the 1500 Hz stimuli. For the 4000 Hz stimuli, the latency for 63 *μ*s was statistically larger than that for 125 *μ*s (*t* = 2.67; *p* < 0.01), which was marginally larger than the latency for 250 *μ*s stimuli (*t* = 1.77; *p* = 0.08). While the latency for 500 *μ*s stimuli was smaller than that for 250 *μ*s stimuli (*t* = 2.87; *p* < 0.01), there was no detectable difference between latencies for 4000 Hz stimuli with 500 or 750 *μ*s ITDs.

Stimulus frequency had a relatively larger effect for ITD than for ILD stimuli. At the smallest ITD magnitude of 63 *μ*s, latencies to 50% correct for 500 Hz stimuli were smaller than those for 1500 Hz stimuli (*t* = 3.93; *p* < 0.001), which were marginally lower than latencies for 4000 Hz stimuli (*t* = 1.84; *p* = 0.07). The same pattern of statistical results emerged for cross-frequency comparisons of latencies to every other stimulus as well. Comparisons between 500 and 1500 Hz reached statistical *p* values below 0.001, whereas *p* values were above 0.05 for comparisons of latencies between 1500 and 4000 Hz stimuli. There were notable individual differences in the ability to perceive envelope ITDs in the high-frequency noises as described in supplementary material 4.^1^

Apart from the latency to 50% correct gaze direction, there were also differences in ultimate success rates for each of the stimuli, seen as the performance level at 1 s post stimulus cue onset (when the correct answer appeared visually). This performance level was modeled by the asymptote of the estimated sigmoidal function, and alternatively calculated more simply by taking the maximum of the averaged data during the time window extending to 1 s post stimulus cue onset. The effects of stimulus magnitude on asymptote were most pronounced for the 4000 Hz ITD stimuli, where there were statistical differences between each of the stimuli (63, 125, 250, and 500 *μ*s), all with *t* values above 2.13 and *p* values below 0.05. For 1500 Hz and 500 Hz stimuli, comparable differences were found only between 63 and 125 *μ*s ITDs.

### C. Measurements of uncertainty: Multiple responses to the same stimulus

**FIG. 4.**
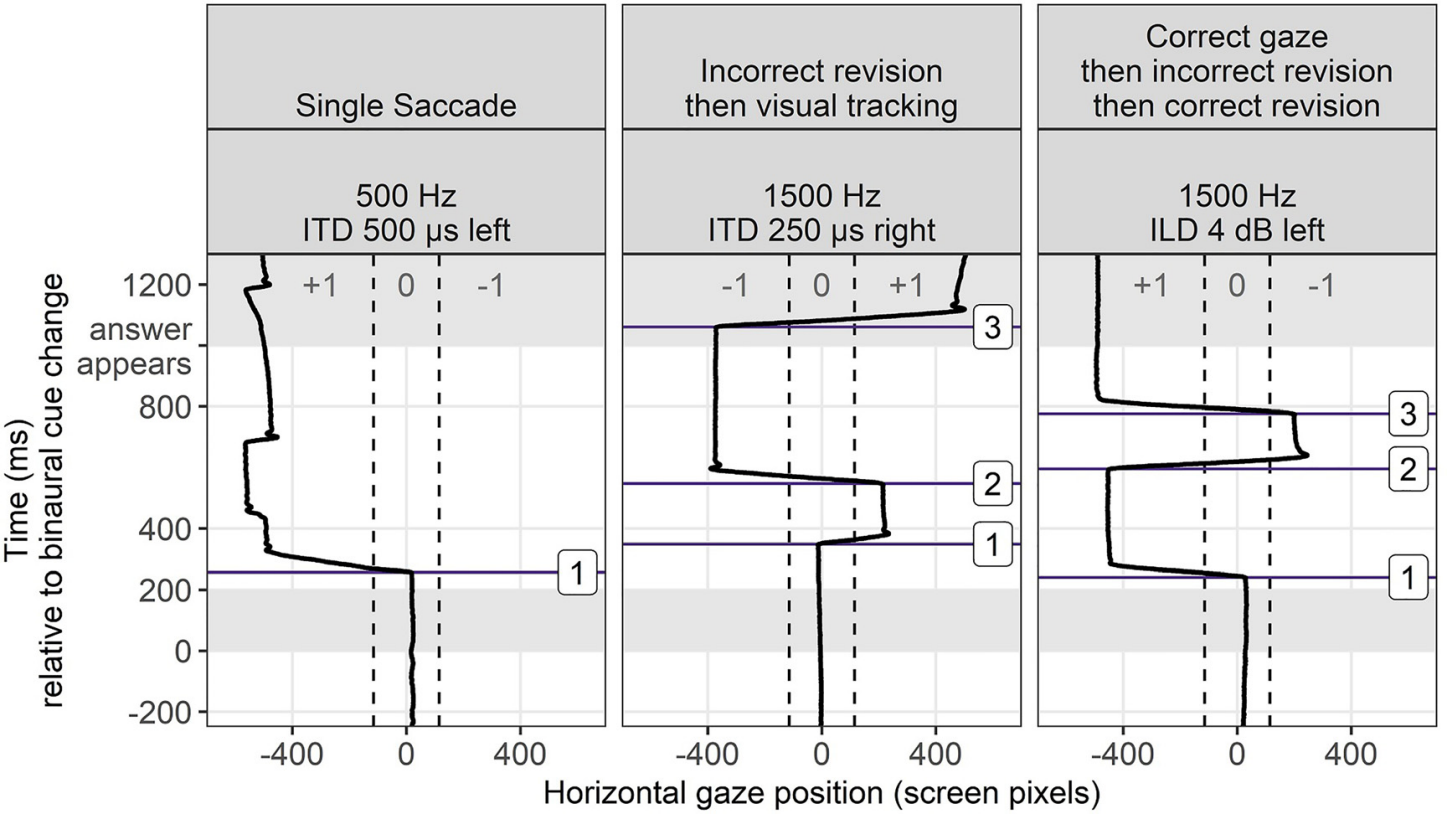
(Color online) Raw data from individual trials showing a single decision (left) or rapid changes in perceptual judgment before landing on a final decision (center and right). Boxed numbers indicate points where a saccade was marked.

One of the advantages of using an eye-tracking method for measuring perception is that eye-gaze patterns change rapidly and are known to update with incremental exposure to auditory stimuli, potentially providing a window into perceptual certainty with granularity not matched by behavioral measures. In contrast to measuring the correctness and reaction time of a “final decision” in a behavioral task involving keyboard, mouse, or button box, we can quantify the change in perceptual judgment over time by inclusion of rapid revisions to that judgment, observed in the eye-gaze data. Figure 4 illustrates some examples of raw data that show the case of a single decision, a decision revised once (before looking at the visual cue to the answer), and a decision revised twice.

**FIG. 5.**
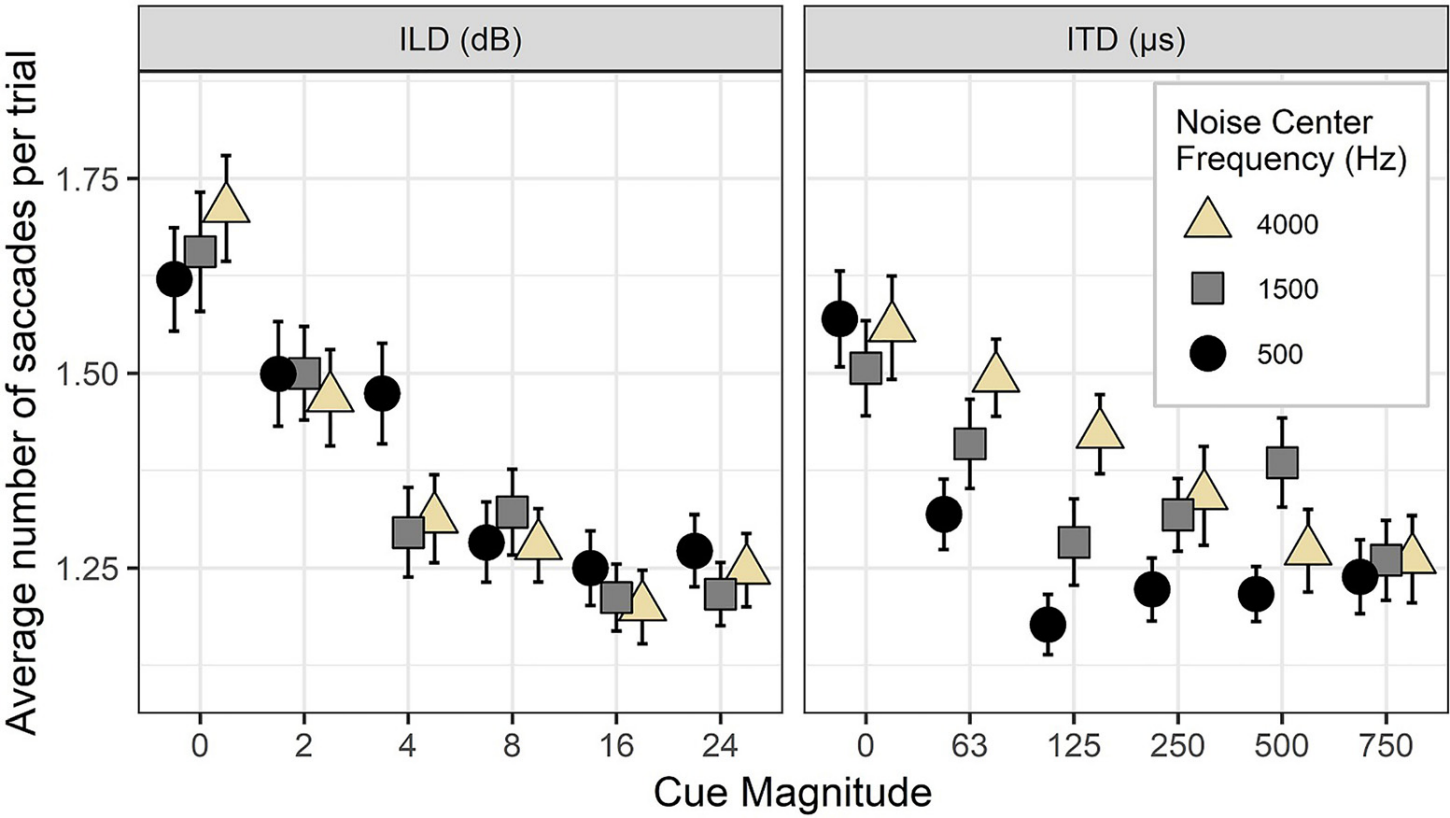
(Color online) Average number of saccades per trial as a function of binaural cue magnitude and noise center frequency. Higher number of saccades is interpreted as evidence of greater perceptual competition or uncertainty. Error bars reflect ±1 standard error of the mean.

There were systematic changes in the number of saccades per trial as a function of stimulus cue magnitude and noise center frequency, illustrated in Fig. 5. As was the case for saccade latency, these data show a monotonic relationship between magnitude and “uncertainty” as measured by increased saccade count. In particular, there were more saccades for ILD cues in general compared to fine-structure (500 Hz) ITD cues, and also more saccades for envelope-ITD (4000 Hz) cues overall. Notably, there were substantial numbers of trials even for “easy” stimuli that elicited multiple saccades, consistent with prior literature on using eye gaze to measure categorical judgments of speech.

ANOVAs revealed significant main effects of cue magnitude and frequency on saccade count for both ILD and ITD stimuli with a significant interaction between these effects for the ITD stimuli. When reducing type I error rate by incorporating random effects of both cue magnitude and frequency, the main effect of frequency in ILD stimuli fell below significance criterion (*p* = 0.07). For ILD stimuli, each cue magnitude elicited a statistically different number of saccades; only for the 4 dB ILD stimuli was there a detectable frequency effect, where 500 Hz noises elicited more saccades (*p* = 0.02). For ITD stimuli, there were a statistically greater number of saccades for 4000 Hz stimuli compared to 500 Hz stimuli for 63 and 125 *μ*s (*p* < 0.01 and *p* < 0.001, respectively).

### D. Distributions of saccade times

Although the average latencies to 50% performance (Fig. 3) tell a clear story about task performance, they also convey the implication that the data are distributed around a single average value, which turns out to be false in some cases. Because multiple saccades can occur in the same trial (Figs. 4 and 5), we examined the timing of all saccades to see if there were concentrations at timepoints other than the overall average.

**FIG. 6.**
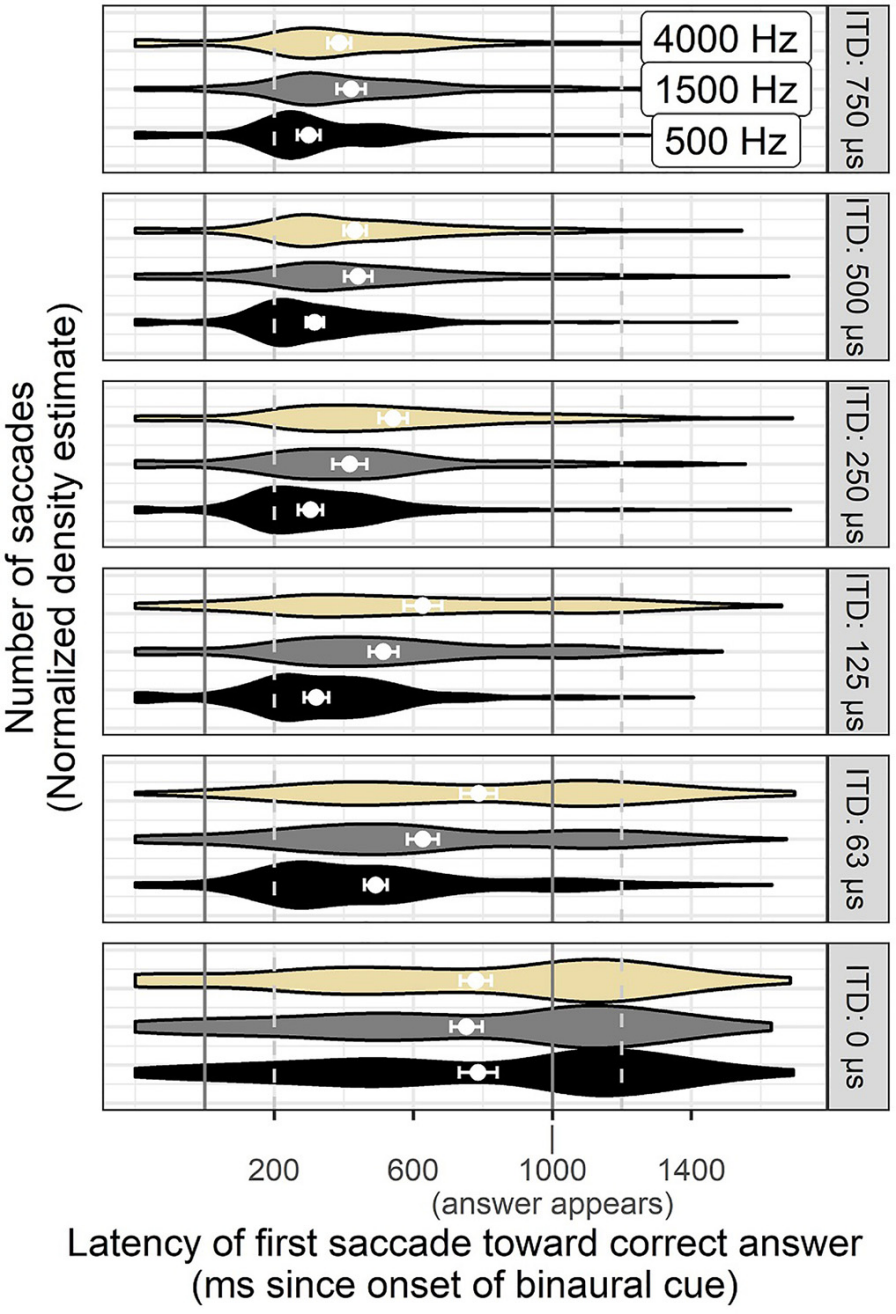
(Color online) Violin plots (symmetrical density plots) of correct saccades observed at each timepoint over time across all ITD magnitudes and noise center frequencies. Greater thickness of the band indicates a greater number of saccades observed at that time. White points with error bars show average latency of saccades to the correct side, which in some cases lies between two concentrated modes of responses. Error bars around the points reflect ±1 standard error of the mean.

Distributions of correct saccade response times for ITD are shown in Fig. 6 where the differences in the distributions were simpler than those for ILDs. Specifically, while responses to 500 Hz (fine-structure) ITDs remain rather tightly clustered at 250 ms for levels between 125 and 750 *μ*s, the responses latencies to the envelope-ITDs in the 4000 Hz noises become gradually more diffuse as cue magnitude becomes smaller (i.e., the violin-density plot becomes flatter in the upper panels).

**FIG. 7.**
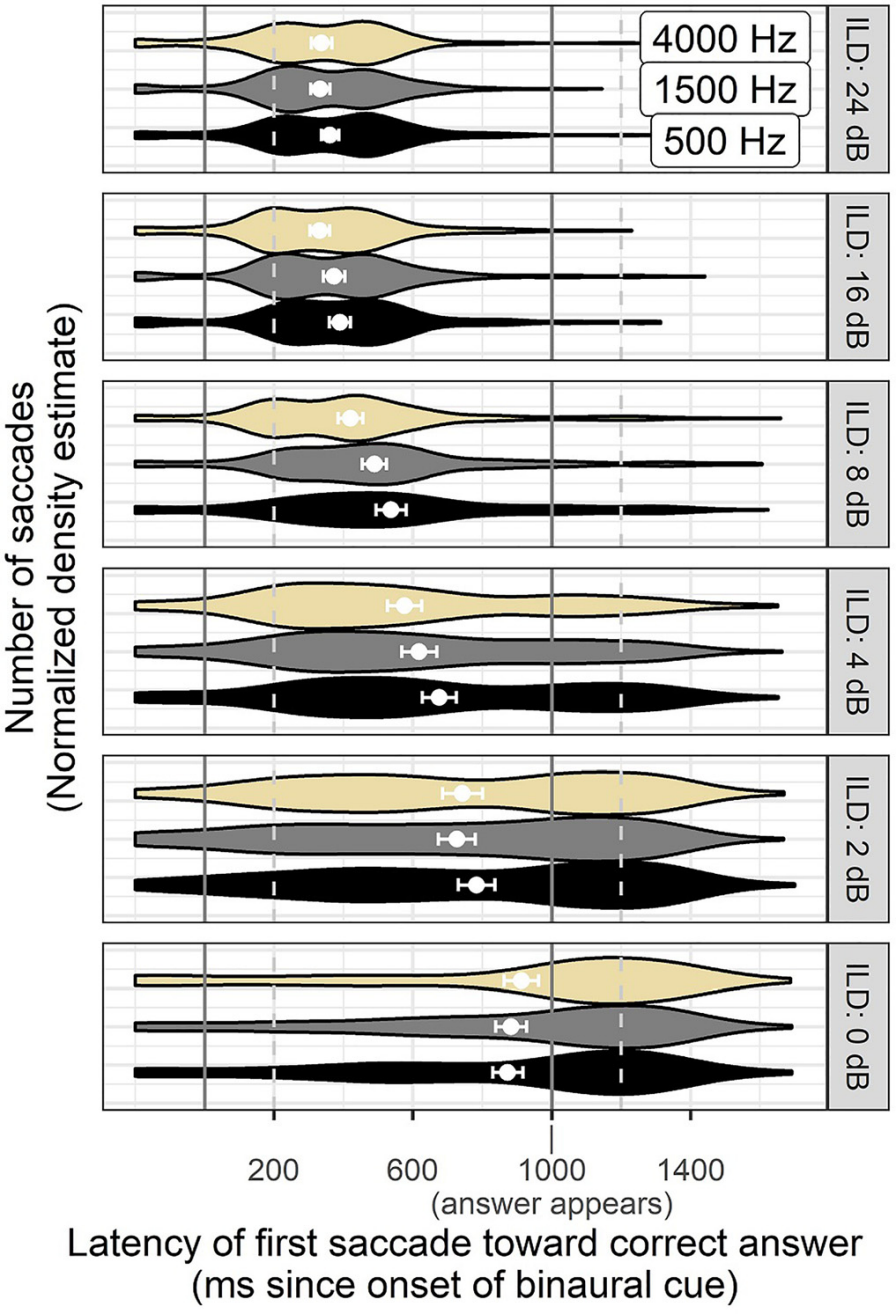
(Color online) Violin plots of correct saccades observed at each timepoint over time across all ILD magnitudes and noise center frequencies. Same as Fig. 6, but for ILD instead of ITD cues. Error bars around the points reflect ±1 standard error of the mean.

**FIG. 8.**
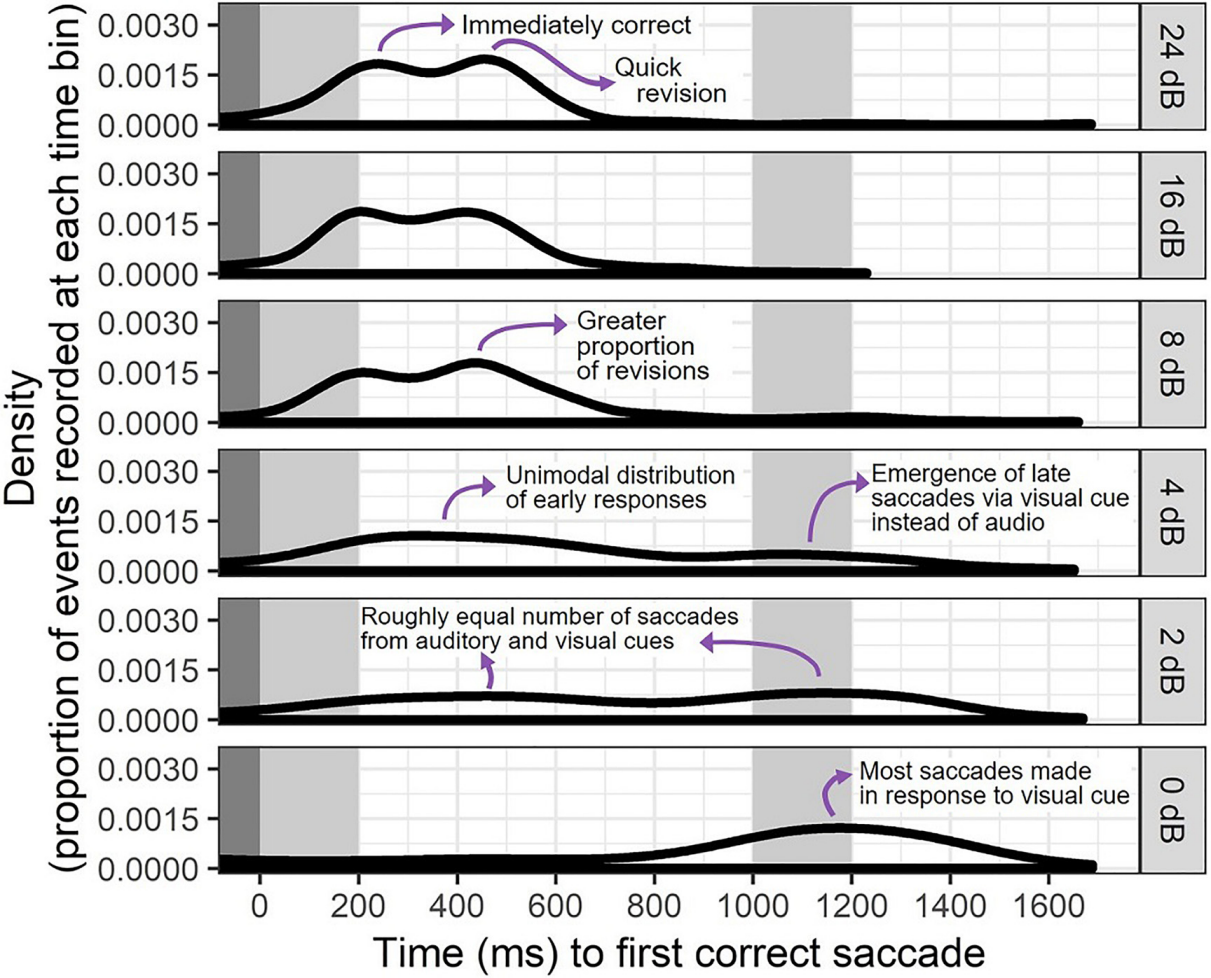
(Color online) Illustration of a possible interpretation of density plot data showing distributions of times to first correct saccade following the onset of an ILD cue in 4000 Hz noise stimuli. Data are a subset of those shown in Fig. 7 with extra detail on the shape and interpretation of the data.

Figure 7 illustrates the distribution of correct saccade times resulting from each stimulus frequency and ILD magnitude. These are the data that contribute to the average latency described above, but in Fig. 7 as for Fig. 6, we see that the pattern of saccade times is not simple enough to be described as a unimodal distribution that shifts to longer latency. Instead, the latency distributions become wider as the stimulus magnitude gets smaller. Numerous stimuli also elicit saccade distributions that are bimodal in nature, possibly because an initial saccade is corrected with a revised one at consistent timing landmarks identified in Fig. 8. Delayed *average* latency for stimuli near threshold could therefore indicate not only a difference in average timing, but also a difference in presence or absence of responses.

Figure 8 shows a detailed look at the change in timing of correct saccades as a function of cue magnitude using the 4000 Hz ILD stimuli as an example. For the largest cue magnitude of 24 dB ILD, there is a bimodal distribution of saccade times with one mode at the earliest expected time of 200 ms post cue onset. The second mode, which comes about 250 *after* the first mode, is interpreted as a sign of corrective saccades that occur as fast as possible following an initial incorrect gaze. Although the presence of incorrect responses at this easy stimulus level might seem counterintuitive, they were less frequent than corrective saccades for higher cue magnitudes, and are generally consistent with earlier literature showing non-zero levels of wrong-size gaze even in the case of unambiguous (speech) stimuli. Later discussion will show that in the cases of double saccades for easy stimuli, the speed of gaze correction was consistent with the ease of perceiving the direction of these stimuli.

The bimodal distribution of saccade times in Fig. 8 is observed in the next two easiest cue magnitudes (16 and 8 dB ILD), but collapses to a broad unimodal distribution when the ILD is only 4 dB. At that cue level, there is also a noticeable number of saccades that occur after the introduction of the visual cue at 1000 ms post auditory cue onset. At the next-smallest cue magnitude of 2 dB ILD, the number of visually guided saccades is roughly equal to the number of saccades guided by the audio stimulus component, indicating a trend that forces critical evaluation of the prior 50% latency data. It appears that at this low cue magnitude, it is not that the latency of auditory-guided saccades is much longer than the latency for stimuli with larger ILDs, but rather that the average is altered by trials where no response was given, since a considerable number of saccades were recorded in response to visual presentation of the answer. The detailed timing information in Fig. 8 is a subset of what is displayed in Fig. 7, and suggests that response timing is a more complicated construct than what could be modeled with a traditional Gaussian or Poisson fit of average reaction times.

### E. Speed of revisions in saccade direction

The bimodal distribution of saccade times for “easy” ILD stimuli (see Figs. 7 and 8) was unexpected. There were common anecdotal reports of a reflexive wrong-way saccade at cue onset that “bounced” into correct position. The substantial number of saccades at roughly 450 ms post cue onset accord with these reports. In question was why any listener would need to revise a decision for a stimulus that should be trivially easy. We note here that the presence of initial wrong-way looks is not incompatible with a task ultimately yielding 100% correct responses, and does not imply that 24 dB ILDs are anything but easy to lateralize for the typical listener. Although it is not possible to fully know the internal decision-making process of the listener, we offer two possible explanations. First, perhaps the consistent timing of the cue led listeners to anticipate needing to make a choice at that moment. They therefore might make an initial wrong guess, hoping to be quick, but then need to make a quick correction. Alternatively, we could recognize that saccades happen rather constantly and it is possible that, even in situations lacking specific uncertainty, observers will “check in” on meaningful landmarks in the visual scene, as would a driver who sees a pedestrian at a crosswalk on the side of the road, and then immediately checks the opposite-side crosswalk entry to check for a pedestrian on the other side. The driver is not uncertain about which side the pedestrian is on, but still scans the visual scene to keep track of important places. In this study, the important landmarks were the ends of the Y-pipe; saccades (both correct and incorrect) were made toward those landmarks.

**FIG. 9.**
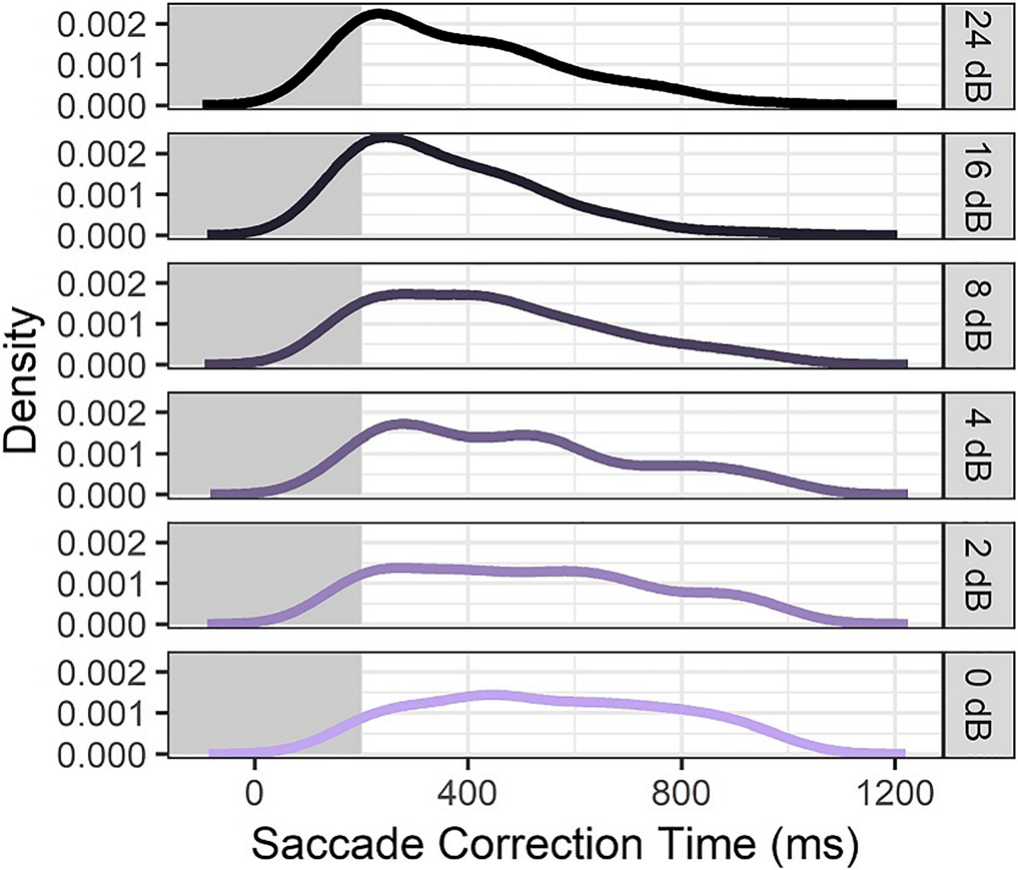
(Color online) Density plots showing distributions of the inter-saccade interval, representing “correction time” for ILD trials where multiple saccades were recorded. Panels show different stimulus ILDs as indicated to the right. Line height represents the relative number of observations across time.

To further explore this pattern of saccade correction and disambiguation here, we analyzed all trials containing at least two saccades and measured the inter-saccade interval as a metric of quickness of revision. It was expected that larger ILDs would result in more rapid revisions in the case of initial incorrect looks because there was stronger information to override the initial wrong decision. For smaller ILDs, on the other hand, inter-saccade intervals should be more uniformly distributed over time since they should reflect more “guessing” rather than “correction.” This prediction was verified by the inter-saccade interval data, which are displayed in Fig. 9. For 24 dB or 16 dB ILDs, corrections were typically as quick as physiologically feasible; for the 2 or 4 dB ILD stimuli, there was much weaker presence of speedy corrective saccades that are observed in easier stimuli.

### F. Behavioral responses to these auditory stimuli

The stimuli in this study were different than stimuli that are used in some classic binaural psychoacoustic studies in that they did not consist of two short intervals of sound separated by an interval of silence. Instead, they were continuous sounds with a change occurring half-way through the stimulus. Additionally, the ILD stimuli were uncorrelated noises rather than correlated signals, motivated by the desire to avoid strong ITD cues that would overpower ILD perception. To obtain some basic performance data for these stimuli, we collected behavioral (i.e., “button-press”) responses for each of the ILD and ITD auditory stimuli (with no animated videos) from an additional 20 listeners (14 women; ages 19–39 yr; all passed 20 dB HL audiometric screening from 250 to 4000 Hz). Participants pressed “F” or “J” (the keys with tactile markers) on a keyboard to indicate left and right responses, respectively. Participants were instructed to respond as soon as they heard the sound shift to the left or right, and were told that the sound change occurred half-way through the stimulus. The auditory stimuli were the same as those used in the videos; the cue change always occurred 2.4 s into the trial. Although there was no accompanying visual cue to the upcoming stimulus change (as in the AEM version of the task described earlier), the cue changes were reliably perceived for the larger cue magnitudes. Response choices and reaction time data were collected using the PsychToolBox suite in matlab, which is designed to maintain excellent temporal precision.

**FIG. 10.**
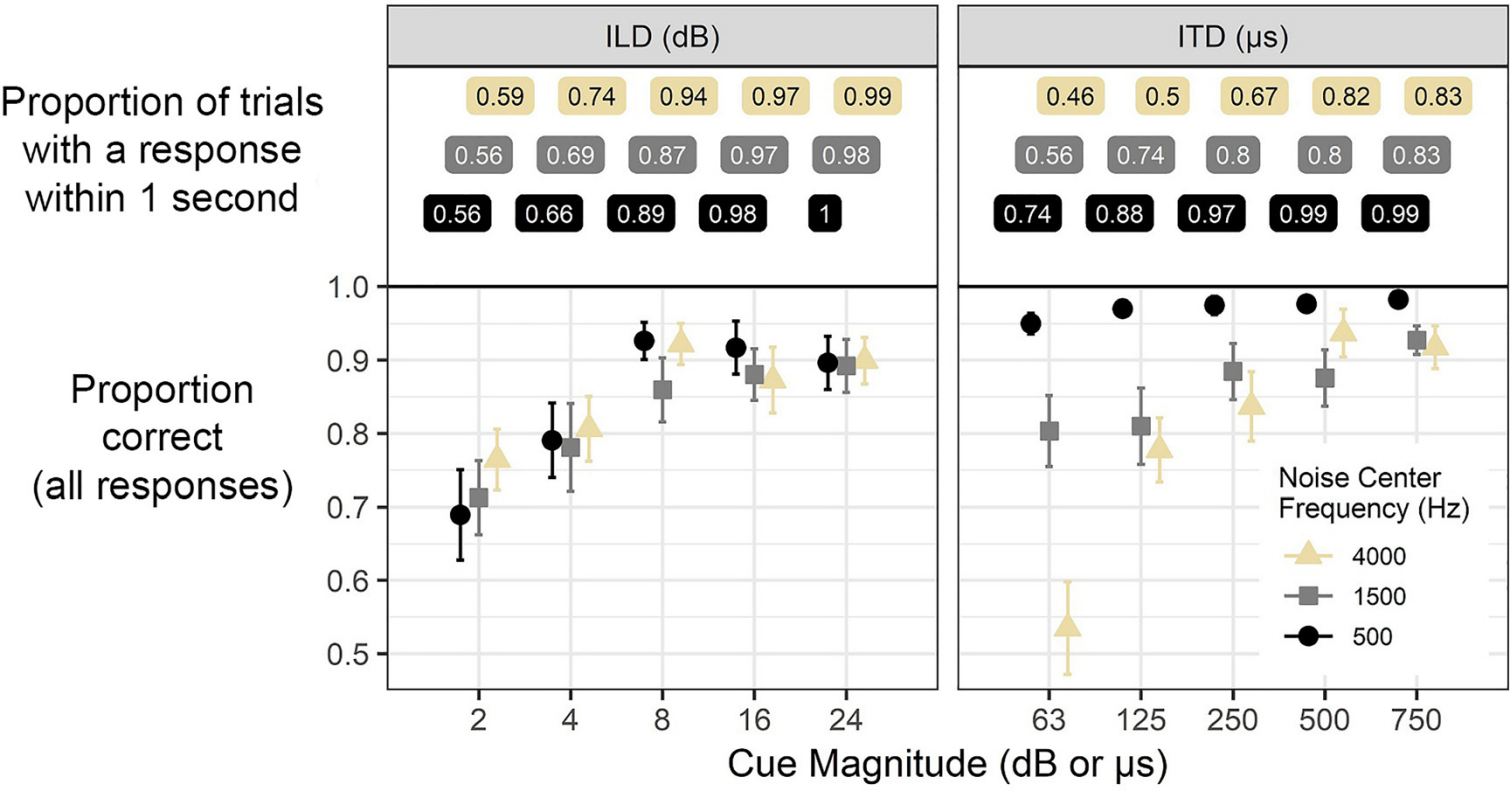
(Color online) Results of binaural cue perception when testing using behavioral button-press methods. Numbers in the top panel indicate the proportion of trials in which any response was recorded within one second of cue onset, which was the window available in the eye-tracking condition before the answer appeared visually. Proportion-correct data reflect all responses, not just those limited by the one-second window. Error bars reflect ±1 standard error of the mean.

Performance data for the auditory stimuli showed that binaural cues of larger magnitude were perceived correctly more often (Fig. 10). With the constraints of limited time to respond and the context of mid-stimulus auditory *change* rather than instantaneous judgment, listeners were not able to achieve *perfect* performance for all stimuli, even if cue magnitudes were larger than thresholds found in previous studies with silence-separated two-interval stimuli.

The top panel of Fig. 10 indicates the proportion of behavioral trials for which there was any recorded response within the 1-s window corresponding to the critical window before the visual target appeared in the eye-tracking experiment; this proportion increases as cue magnitude rises, and also shows a dependence on stimulus frequency that is consistent with the eye-tracking data. Low-frequency noises elicited more responses than high-frequency noises for ITD, and there was a very slight reversal (or at least neutralization) of that trend for ILD. It should also be noted that for a considerable portion of trials with small cue magnitude, listeners would frequently fail to initiate any response at all within that 1-s window, suggesting that although the cue initiated saccades, it might not have been enough for a participant to commit to a motor response. This pattern suggests that the early-occurring saccades in the main experiment might have been precursors to behavioral responses and therefore not measurable with behavioral methods. However, the two tasks did not have identical constraints on the response window so a firm conclusion is hard to draw.

**FIG. 11.**
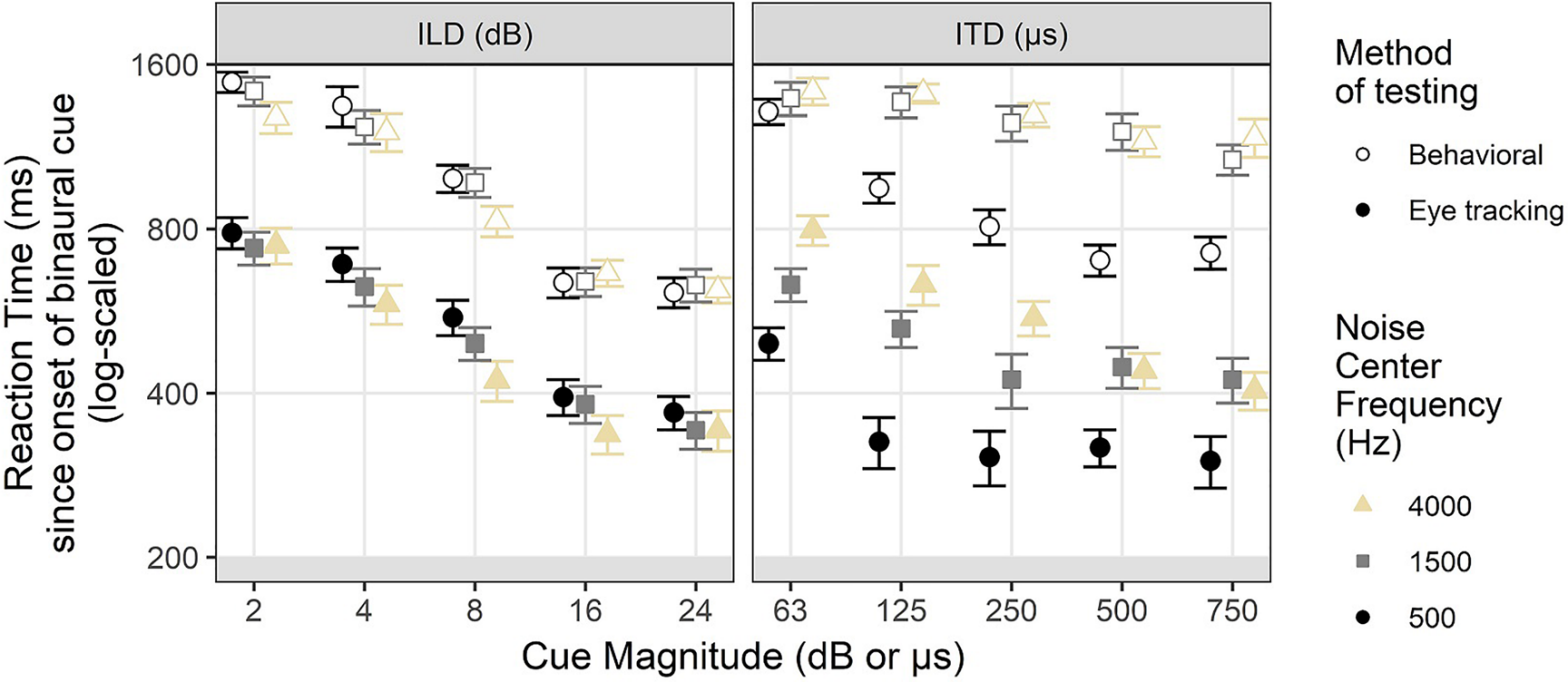
(Color online) Comparison of log-scaled reaction times for correct responses collected via button-press responses (open shapes) and saccades (filled shapes) for all stimulus parameters for both binaural cues. Error bars reflect ±1 standard error of the mean.

Behavioral reaction time is compared to saccade reaction time in Fig. 11, which shows data for correct responses only. Behavioral and saccade responses were systematically faster for binaural cues of larger magnitude for both ILD and ITD. Saccades were quicker overall (by 200–700 ms for ILD and 600–800 ms for ITD) compared to behavioral responses for both cues at all frequencies and all cue magnitudes. Saccade reaction times generally showed a subtle but orderly relationship across frequency (higher frequencies elicit faster saccades for ILD and slower saccades for ITD). That relationship was somewhat more consistent for the eye-tracking results, as the behavioral results showed occasional reversals of the effect direction or were undifferentiated for some stimulus magnitudes. The low-frequency ITD noises elicited much faster reactions times regardless of response collection method.

## IV. GENERAL DISCUSSION

Eye movements can be guided by binaural hearing with timing and patterns consistent with previous studies using the AEM paradigm (e.g., McMurray and Aslin, 2004). In this study, the binaural cues were embedded in a change-detection task rather than the instantaneous judgment task used in many previous psychoacoustic experiments. Successful saccades were elicited more quickly in response to ILD or ITD cues of higher magnitude (Figs. 2 and 3), suggesting that the method is sensitive to saliency of cues, not merely whether they are detectable in an all-or-none fashion. Participants occasionally revised their gaze direction before resting on a final decision (see Figs. 4 and 5), even in the case of stimuli thought to be trivially easy. It is feasible to interpret the number of fixation changes as a proxy measure of uncertainty. The distribution of saccade times was not always unimodal (Figs. 6, 7, and 9), indicating separate clusters of response times for initial responses, revised responses, and responses driven by visual cues.

### A. Comparison with behavioral methods

Although there was arguably less granularity in the behavioral data with respect to the effect of stimulus frequency, the behavioral method proved to provide a good metric of overall performance with sensitivity to suprathreshold cue magnitude in both the performance (percent correct) and reaction time results. Missing from the behavioral data was the measure of the time-course of perception, including revisions, or any account of perceptual competition. Additionally, the lack of behavioral responses within 1 s (at least for more difficult stimuli) suggest that intermediate stages of perception were detectable in the saccade data.

Considering the importance of binaural hearing in the navigation of everyday auditory scenes, the study of perceptual uncertainty rather than only perceptual accuracy and acuity might be a long-overdue subject of investigation, particularly for hearing impaired populations that may have more difficulty processing binaural cues and show a tendency to wait longer before initiating responses (McMurray *et al*., 2017).

Behavioral data and eye-tracking data both showed low success rates for ILDs of 2 and 4 dB, which are typically considered to be reliably perceptible by typical listeners. However, this pattern is explained somewhat by the use of continuous sound rather than static intervals of sound separated by silence. The binaural cue change in this study was in the *middle* of the stimulus, where Stecker and Brown (2012) showed sound changes are less perceptible than those at the onset or offset. A silent gap interval between cue levels—as used in many other classic binaural studies—could potentially “restart” the system (Hafter and Buell, 1990) or let the effects of the first interval (zero-level binaural cue) dissipate before perception of the relevant cue. Another factor that likely contributed to poorer ILD performance in this task was that the noises were uncorrelated, which is a factor discussed in Sec. IV B.

### B. The effects of noise frequency, envelope fluctuations, and interaural correlation

This study unexpectedly showed an apparent frequency dependence for ILD cues; listeners responded more quickly to ILD cues in high-frequency noise than in low-frequency noise (Figs. 2 and 3). Although some studies have established poorer ILD perception for specific frequencies (Yost and Dye, 1988; Goupell and Stakhovskaya, 2018), the apparent monotonic advantage for higher-frequency noises was not expected. However, it is possible that “frequency” was actually not the operative factor affecting the results in the current study.

Stimuli that differed by frequency also differed by envelope modulation strength because 1/3 octave corresponds to different linear bandwidths across different center frequencies. The 4000 Hz noise had the smoothest amplitude envelope because a wider range of random-phase frequency components could neutralize amplitude fluctuations by filling in the gaps in the envelope. Conversely, the 500 Hz noise had fewer linear components, and therefore had numerous notable momentary peaks and valleys in intensity. The reduced success rate for 500 Hz stimuli might have therefore been affected, at least partially, by the greater moment-to-moment intensity fluctuations in each ear. Incidentally, the advantage for perceiving ILD for 4000 Hz compared to 500 Hz noise bands observed by Grantham (1984) might have also been affected by the choice to use stimuli equalized for octave-scaled bandwidth rather than linear bandwidth.

In addition to differences in uncorrelated envelope *fluctuations*, there were binaural envelope *correlations* that differed across noise frequencies as well. The envelope correlation, described in detail by van de Par and Kohlrausch (1998) and Bernstein and Trahiotis (2002), was at a maximal level of 1.0 for ITD stimuli. For ILD stimuli the envelope correlation was reduced for low-frequency noises because the non-correlated differences between ears were magnified by greater fluctuations. As such, the 500 Hz noise also had more dramatic changes in instantaneous ILD, which could explain the reduced success rate. Details of the instantaneous ILD and average normalized interaural envelope correlation are illustrated in supplementary material 5.^1^

The impact of interaural decorrelation suspected *post hoc* in this paper has precedence in the literature. Hartmann and Constan (2002) found that decorrelating left and right channels of an ILD stimulus elevated discrimination thresholds, although only very slightly. Brown and Tollin (2016) corroborated this observation with neural recordings in the chinchilla, and found weaker perception of uncorrelated stimuli by humans, even if perceptual lateralization remained relatively unaffected. Laback *et al.* (2017) proposed a neural model that would account for temporal effects in both ILDs and monaural sequential level differences and found that, among other results, the model successfully accounted for the effects of interaural decorrelation identified by Hartmann and Constan (2002). Laback *et al.* (2017) suggest that amplitude modulation enhances ILD sensitivity on the basis of better sensitivity for pulse trains compared to pure tones. This leads to the reasonable prediction that correlated modulations should promote better sensitivity, which is consistent with the coincidence framework described by Ashida *et al.* (2016), which predicts noteworthy influence on excitatory inputs on the basis of coincidence specifically. Motivated by earlier studies that examined the role of envelope fluctuations on ILD perception (e.g., Goupell, 2012; Stecker, 2016), ongoing work in our lab continues the paradigm described in this study, using stimuli with controlled amounts of inherent fluctuations within the same narrow spectral band and with envelope correlation explicitly controlled.

### C. Other applications

There is potential diagnostic utility in the method described in this paper. In cases where a person is suspected of being at risk for poor binaural hearing, a sensitive and reliable method is needed to detect abnormalities. There are populations suspected of having deficient auditory systems, such as people with head trauma and blast exposure (Gallun *et al*., 2012), older listeners (Füllgrabe *et al*., 2018), as well as people who use bilateral cochlear implants, which provide input to both ears, but in a way that is not synchronized (Kan and Litovsky, 2015). In each of these cases, binaural perception might be affected not only by poorer accuracy or threshold-level acuity, but also by uncertainty for suprathreshold hearing. Current methods of detecting binaural deficits include batteries of perceptual tests such as the binaural masking level difference and staggered spondaic words (Gallun *et al*., 2012), adaptive tracking of highest frequencies for which binaural disparities in temporal fine structure is detected (Füllgrabe *et al*., 2018), and classical tests like sound localization and ITD discrimination. The current method could be useful in specific cases where one might expect that final responses might not indicate the course of uncertainty resolution that might exist in a person's perception. Additionally, since this method has been used in infants as young as six months of age, it might be possible to obtain accurate measures of binaural hearing in young listeners for whom classical psychoacoustics is not feasible. One could measure saccade latency to obtain a rather precise estimate of when perception changed, while avoiding some of the complications of motor control timing and ability.

Another application that might naturally come to mind when considering the current technique is the potential application to visually guided hearing aids (Kidd *et al*., 2013). Although the current study used eye movements as *responses to* rather than *initiators of* sound changes, the connection is apparent. The dynamics of eye movements in response to perception of sound location hold potential value, especially in cases where vision might drive auditory attention in *dynamic* listening situations (e.g., Best *et al*., 2017). The current study shows that binaurally-guided eye movements can change rapidly in as brief a time as 800 ms, suggesting that visually guided directionality changes might not work optimally if they act upon *every* saccade, but rather integrate across time.

There is potential for various alternations of the current experiment design if an experimenter has specific goals including (1) adaptive methods, (2) the two-interval silence-separated stimulus design to compare against thresholds obtained with popular behavioral methods, (3) where the stimulus is immediately lateralized after onset silence in order to see if performance would improve when avoiding carryover effects from the front-cueing first half, and (4) randomizing the amount of time before stimulus cue change to remove the predictable timing. Given the numerous potential contributors to certainty and decision-making regarding binaural perception, the current method could prove useful to probe perception in multiple ways.

### D. Note on analysis and coding of data

One of the planned analyses in this study was conventional binomial logistic regression where correct saccade directions were coded as 1 (hits) and center/incorrect gaze fixations would be coded as 0 (misses). The advantage of this approach is that it incorporates well-established techniques of binomial logistic regression, including mixed effects modeling. However, there were multiple reasons why this analysis approach was abandoned. The first disadvantage of this type of regression is the assumption that data eventually asymptote at 1; this assumption turned out to be clearly not suitable for lower-magnitude ILDs and some ITD levels with 4000 Hz noise (see Fig. 2). The other disadvantage is that the constraint of 0 and 1 as outcome measures means that looks to the *wrong* side are treated the same as looks that remained at center. In other words, this approach does not capture the difference between making a wrong decision and having made no decision at all. The analysis used in this study for modeling success rate over time was chosen partly to ensure that looks to the wrong side were penalized rather than ignored.

## V. CONCLUSIONS

Eye tracking can be a sensitive way to assess temporal aspects of binaural hearing, including the rapid accumulation of certainty and incremental perceptual decision-making and revisions as a listener is exposed to an auditory stimulus. The current method offers some advantages over conventional psychoacoustics behavioral methods that might be relevant for particular problems that demand granular measures of certainty or measures with extra sensitivity in the time domain. Specifically, the number and timing of revisions in perceptual judgment appear to correspond with stimulus attributes that should scale with certainty, as observed in prior literature using speech stimuli. Although these advantages are not central to the goals of every binaural hearing experiment, they could play a role in the exploration of psychoacoustic perception that parallel the meaningful progress made in language processing. Specifically, we are able to assess how perception changes moment-to-moment as saccades indicate perceptual judgments that are considered before a final decision is rendered. This allows for quantification of perceptual competition and, possibly, time-locking saccades to specific stimulus events more tightly than what can be achieved through physical responses.

The gaze patterns elicited in this study reflect gradient sensitivity rather than all-or-none perception determined by threshold. Response to ITDs is faster and more reliable when driven by temporal fine structure in low-frequencies compared to envelope cues in the high frequencies. Responses to ILD cues in high-frequency noise were slightly earlier than those in low-frequency noise, potentially because of differences in envelope fluctuations or interaural correlation. Regardless of the reason for the latency difference, the effect of stimulus frequency was captured in the eye movements more reliably than in the timing of behavioral (motor) responses, perhaps because of the relatively more complicated musculature involved in motor movements and individual variability in motor speed. Finally, we emphasize that measurement of auditory processing as it unfolds moment-by-moment (rather than just end-point performance) could be a tool that unlocks previously hidden aspects of perception and potentially motivates further creativity in the assessment of binaural hearing and audition in general.

## Footnotes

^1^ See supplementary material at https://doi.org/10.1121/1.5088591 E-JASMAN-145-004902 for details of the coding of raw gaze data, modeling of ILD and ITD success rate, individual differences of envelope ITD perception, and quantification of the interaural correlation of the ILD stimuli.
